# 

**Published:** 1876-05

**Authors:** 


### TABLE OF CONTENTS.

ORIGINAL COMMUNICATIONS—

Texas State Medical Association, by A. R. K. .... 257
Case of Opium Poisoning, by Dr. A. R. Alley, Atlanta, Ga .... 268
Case Reported by D. C. Wilson, M.D., of Ironton, Ohio .... 269

SELECTIONS—

International Medical Congress .... 270
Rupture of the Urethra. By J. B. Hamilton, Assistant Surgeon U.S.A., Fort Colville, Washington Territory .... 272
Treatment of Fistulous Ulcers .... 275
Concentrated Vegetable Medicines. By A. G. Stallnaker, M.D., Walton, West Virginia. 278
On the Origin and Treatment of Purulent Ophthalmia .... 281
General Rules in Operating with the Galvanic Cautery. By C. T. Deane, M.D .... 283

EXTRACTS AND GLEANINGS—

Ulcers, 285. Burns, 286. Diseases of the Uterus, 287. A Modification of the Operation for Phymosis, 288. Prevention of Miscarriage by Hypodermic Injection of Morphia, 289. On the Indications Against Version in Shoulder Presentations, 291. Bryant's Test Line—Its Diagnostic Value in Cases of Injury of the Hip Joint, 293. Digitalis in Typhoid Fever, 294. Pericarditis; Treatment by Means of Ice-Bags, Ibid. Gallic Acid in Albuminuria following Scarlatina, 295. Plugging the Nasal Cavities. 2[illegible]5. Anæmia, 296. Solution of Bromine as Dressing, Ibid. Nitric Acid as a Caustic in Uterine Practice and its Superiority as such to Nitrate of Silver, 297. Pills for Obstinate Neuralgia, 297 A Certain Cure for Rheumatism, 298. Typhoid Fever Probably Caused by Infected Milk, 299. Iodide of Starch as an Antidote to Poison, 299. Treatment of Orchitis, 300. Treatment of Dysentery with Salicylic Acid, 300. Hip Disease—Its Treatment, 301. Chloral Suppositories 301. Tinea Decalvans, 302. Salicylic Acid for Offensiveness of Breath and Expectoration, 302 Rupturing the Membranes, 303 Catherteism in Laceration of the Urethra, 303. An Improved Method of Obtaining Support for Fractured Bones of the Extremities, 304. Electricity in Diseases of the Skin, 304. Digitalis, 305. Chloro, in the Surgery of Infants, 305. A New Use for Carbolic Acid—Opening Abscesses without Pain, 306. Chlorate of Potassa in Vaso-Paralytic Diarrhœa, 306. Caustics in the Treatment of Diseases of the Cervix, 306. Grindelia Robusta—Its Medical Value, 307. Bromide of Lithium, 307. Ulcerated Nipples, 308. Bromide of Camphor—Its Therapeutic Value, 308. Ergot of Rye as an Antipyretic, 308. What Vaseline Is, 309. Chloroform as a Preservative Agent, 309. Ergot for the Intestinal Hemorrhage of Typhus, 309. Chlorine Water in the Treatment of the Injuries Received from Rabid Animals, 310. Intestinal Hemorrhage in Typhoid Fever, 310. Treatment of Burns, 310. Salicylic Acid for Foul Breath and Offensive Expectoration, 311. How to Make Raw Meat Palatable to Invalids, 311. Parametritis and Peri-Metritis, 311. Salicylic Acid in Acute Rheumatism, 312. Treatment of Scrofulous Sores or Runnings, 312. Dressing for Burns, 312. Jervia—A New Alkaloid in Veratrum Viride, 312.

EDITORIAL AND MISCELLANEOUS—

Georgia Medical Association .... 313
Medical Societies and Organizations .... 315
Important Item—Medical Education .... 317
Transactions of Medical Association of Alabama, 1875 .... 319
Doctors of Pharmacy .... 319
State Board of Health .... 320

